# Medical Opinion and Movement

**Published:** 1910-07-09

**Authors:** 


### Medical Opinion and Movement.

### Differential Radio-diagnosis of Calculi.

It is generally recognised that whereas radiography is usually successful in revealing the presence of renal calculi, this is not the case with gall-stones. Dr. Béclère records a case, however, in which the $x$-rays showed the presence of a gall-stone instead of a supposed renal calculus in the right kidney. In order to distinguish the radiograph of a gall-stone from that of a right renal calculus, Dr. Béclère advises that two successive radiographs be taken of the right side. The patient should lie on his side, and the one radiograph is taken with the plate under the lumbar region and the tube above the hypochondrium, and the other with the position of plate and tube reversed. Care must be taken that the focus of the rays is at the same distance from the plate for both radiographs. Under these conditions the radiographic image of the stone will be larger the greater its distance from the plate. If it is larger on the lumbar plate than on the plate placed over the hypochondrium, it is a gall-stone and not a renal calculus. According to this author this method of examination should always be carried out where radiography shows the presence of a stone on the right side of the abdomen in order to exclude the possibility of it being a gall-stone instead of a renal calculus.

### Dioxydiamidoarsenobenzol in Syphilis.

Professor Ehrlich has endeavoured to discover by an extended series of researches an arsenic compound which would be able to destroy at once, if possible, by one injection the trypanosomes of disease. Such a substance must, of course, be at the same time highly toxic for the parasite, and only feebly so for the subject injected. Ehrlich and Hata believe they have obtained such a substance in the organic compound of arsenic to which they give the name dioxydiamidoarsenobenzol. At a recent meeting of the Berlin Medical Society the efficacy of this new drug in the treatment of syphilis was fully discussed by Wechselmann, Michaelis, and others, and so far the results obtained appear to be highly satisfactory. The drug may be administered either by intravenous or intramuscular injection, preference being generally given to the latter method, and the usual dose is twenty-five to fifty centigrammes. The injections are accompanied by more or less local pain, and not infrequently followed by a slight rise of temperature. Dr. Wechselmann's experience extends over eighty cases in the course of three months, and he is convinced of the specificity of the drug, which, according to him, acts with a certainty and rapidity against all primary, secondary, and tertiary symptoms superior to any other form of treatment. With one injection of a comparatively small dose he has seen the complete disappearance of the most severe symptoms, which had previously proved refractory to mercurial inunctions and injections. In view of the action of other arsenic compounds upon the optic nerve, care was taken to exclude from treatment any case already affected with optic neuritis. By mistake one such patient received the treatment. In this case, however, the treatment did not cause any further change. The evidence afforded by Michaelis and others was equally favourable, but it was generally agreed that time must elapse before the possibility of recrudescence of the disease could be determined. Similarly successful results with the treatment are also reported by Dr. W. Pick and others at a meeting of the Vienna Medical Society.

### Palpation of Arteries by the Finger-nail.

In determining the condition of the arteries it is usual to palpate the arteries with the pulp of the finger. According to Dr. Wortheim Salomonson much more definite indications are obtained by employing the finger-nail. For this purpose the nail should be glided either across or along the artery. By means of the nerve filaments in the nail matrix the pulsations are easily felt, and when these cease by means of compression the artery itself is well defined, and by careful palpation in this way the thickness, irregularities, and sinuosities of the arterial walls can be well appreciated. The author explains the greater delicacy of manipulation in this way by the fact that at a given moment the sensations obtained are very limited instead of being diffused over a large surface, as in the case of palpation with the finger pulp. Moreover the resistance of the vessel, which is felt as the finger-nail passes over its border, is instantaneous, and in consequence the perception is much more definite and precise. All the superficial arteries can be palpated in this way, and according to this author it is especially useful in the palpation of the posterior tibial, and of the arteries of the foot in cases of intermittent claudication. On the other hand, large vessels, such as the carotid and femoral arteries, do not rest upon a sufficiently resisting bed for this method of examination.

### Treatment of Local Suppurations.

It may be remembered that at the last German Surgical Congress, Professor E. Müller, of Marburg, brought forward his ferment and anti-ferment treatment of local suppurations. The treatment is based upon the relations of the proteolytic ferment of the polynuclear leucocytes, and its antagonistic anti-ferment in the blood serum and in serous exudates. The pus cells of acute suppurations are rich in this albumen-dissolving ferment, and thereby cause destruction of tissue. On the other hand, tuberculous pus appears to be deficient in this enzyme, and Müller seeks to increase absorption of a tuberculous exudate by the addition of this ferment. The anti-ferment treatment is applicable to ordinary acute suppurations in which it is sought to inhibit the destruction of tissue by injection of the anti-ferment. The anti-ferment may be obtained in various ways, but the method found most practical consists in employing serum from animals immunised against pancreatic trypsin. The pus is first evacuated by aspiration, or by a small incision, and the serum is then injected into the abscess cavity. Gergö reports, in the *Deutsche Zeitschrift für Chirurgie*, observations on the treatment in 160 cases. He commends it for local suppurations, including fistulæ, and finds that it produces rapid healing and better cosmetic results than ordinary incision. He had no evil consequences in the way of serum rash or anaphylactic reaction. In the same journal Brüning publishes the results of treatment in over 100 cases, which appear to be equally favourable, but he records one death, which he attributed to anaphylaxis. In this country David McEwan has tried the method in fifteen cases with satisfactory results. On the other hand Dr. Hirsch, of Vienna, condemns the method as theoretically unsound, and in practice he finds it useless and of no more value in the treatment of abscesses than simple puncture. Professor Müller suggests the use of the treatment to inhibit the destruction of cerebral tissue after opening a brain abscess, and also in cases of empyema, suppuration, peritonitis, and the like.

### Infectivity of Scarlet Fever.

A valuable note on a remarkable case of scarlet fever occurring in one of the remotest parts of the Empire is contributed to the *Lancet* by Major Hunter, of the Indian Medical Service. The patient was a forest officer stationed in a particularly out-of-the-way part of the Sub-Himalayan Terai in India, and he had no communication with the outside world for many months except by letter. He suffered a typical attack of scarlet fever. When this diagnosis was conveyed to him it was remarked that it was a mystery how he contrived to acquire the disease. The explanation was then given by the patient himself. By the preceding mail he had received from Scotland a letter from his younger brother telling of an epidemic of scarlet fever at school, on account of which he (the brother) had been sent home. The letter was written on the home notepaper, which, presumably, had not been at the school, and the writer of the letter had not had the disease and did not develop it. Yet, apparently, the contagion was transmitted from one of the affected schoolboys through an uninfected individual, and was able to set up the disease in a susceptible individual as far away as India. If this actually was the sequence of events, as seems possible, it proves how formidable a task the suppression of infectious fevers by isolation is.

### Errors of Diagnosis.

At the recent meeting of the American Medical Association Dr. R. C. Cabot delivered an address on the study of mistaken diagnoses based upon a thousand autopsies. Accurate statistics showing the percentages of error, both in commission and omission, in all kinds of local and general diseases were presented. Among the most important and most practical of the conclusions drawn from these figures are the following: Never make a diagnosis of uræmia in a patient seen for the first time, comatose or convulsed; such diagnosis is rarely right. Ptomaine poisoning should be diagnosed only with definite chemical evidence; peritonitis and tabes are more likely to be correct explanations of the symptoms. After 45 years of age arteriosclerosis, hyperthyroidism, dementia paralytica, and pernicious anæmia are much commoner than hysteria or psychoneurosis. Diagnoses of tertian malaria when the symptoms resist quinine for more than three days are almost invariably wrong. Typical migraine is often a symptom of unrecognised brain tumour or of chronic nephritis. Most cases of bronchitis are really tuberculosis, broncho-pneumonia, or multiple bronchiectatic cavities. Apart from the immediate results of the acute infections, such as scarlet fever, diphtheria, tonsillitis, and pneumonia, acute nephritis usually turns out to be chronic. Acute gastritis and gastralgia usually mean appendicitis, gall-stones, or peptic ulcer. Pus in or near the liver is often mistaken for a serous or purulent pleurisy, for it produces identical signs in the chest posteriorly.

An *x*-ray picture of the shinbones may give the first hint of an active syphilitic process in the joints or internal viscera. Systolic or presystolic murmurs heard best at the apex of a markedly enlarged heart rarely mean valve lesions. Diastolic murmurs at the base of the heart afford very uncertain evidence of aortic disease unless there are characteristic jerkings in the peripheral arteries. Myocarditis is a diagnosis which can never be made with any certainty. Besides the direct evidence afforded by the history and physical and chemical examination, diagnosis profits much by taking account of certain familiar pathological chains or groups of items; given one or two members of a group, it is often wise to act as though the others were present, provided, of course, that direct evidence in no way contradicts this assumption. Cerebral localisation applied to tumours, hæmorrhages, and the like, is still in its infancy. The clinical diagnosis of so-called diseases of the blood is still the easiest and surest in medicine. Startling as some of these generalisations may appear, it is to be remembered on what a wealth of clinical and post-mortem experience their author draws, though it is also easy to grasp that such conclusions founded on deadhouse statistics alone contain distinct elements of fallacy. Thus, for example, it is very likely true that most *fatal* cases of bronchitis will display broncho-pneumonia, bronchiectasis, or tuberculosis; but that is because these common complications of simple bronchitis are so very much more likely to prove fatal than bronchitis itself.

### Treatment of Eclampsia.

That uræmia, or eclampsia, is caused by the retention in the blood of the salts (principally sodium chloride) which the damaged kidneys are unable to throw out in sufficient quantity is the conception upon which Dr. Jacobson bases his treatment for eclampsia, described in the *American Journal of Obstetrics.* Since retention of salts raises the molecular concentration of the blood, and at the same time its specific gravity, he aims at reducing this concentration to somewhere near the normal; when this is accomplished the patient will, he says, at least temporarily recover. For securing this object there are only two available methods: starving the patient and diluting the blood by adding water. In pursuit of the former object he withholds all food for three days, allowing nothing but water, plain or aerated. This is inadequate as a means of diluting the blood, because comatose patients cannot drink and those who are not comatose may refuse it. So the author resorts to rectal injections; he finds that plain water creates irritation, and normal saline is inadmissible as illogical and dangerous. Sugar was selected as the best substitute for salt on account of its high molecular weight. Continuous rectal irrigation by the drop method with sugar solution (strength not stated) has been tried with marked success in several cases, two of which are reported in full, and the specific gravity of the blood falls markedly during the treatment. The other common methods are not neglected, however; veratrum viride is given hypodermically, hot packs and purges are freely ordered, and later on a salt-free diet is prescribed. It is suggested that the treatment is harmless and worthy of further trial, but the author wisely avoids premature claims to the discovery of an infallible remedy for this often intractable disease.

### Trauma to the Kidney.

According to Noever in the *Annales de la Société Belge de Chirurgie*, the true capsule of the kidney is almost always ruptured when the organ is subjected to severe injury. The fatty capsule, however, usually remains intact, despite the extravasation into it of blood, and in some cases of urine as well. Hæmorrhage, as a rule, soon ceases, though an enormous hæmatoma may be formed if the vessel torn be a large one. This tumour may become gradually organised and a spontaneous cure result, but in some cases infection takes place and a peri-renal abscess results. Or again, the tumour may persist, and, acting as a foreign body, excite an inflammatory reaction in the surrounding tissues, with the formation of adhesions to the diaphragm, peritoneum, and colon. If the extravasation be mainly urinary, a traumatic swelling of cystic nature may follow, which will burst later into the pelvis of the kidney, or, if the ureter be occluded, may persist, as in one of the author's cases. In all cases operative measures should be undertaken at once, and these should aim at preserving the parts as far as possible. Ruptures in the kidney substance should be sewn up, even if the tear is extensive. If operation is unavoidably delayed for some time after the injury it is inadvisable, owing to the dense adhesions which are always formed in cases of this kind, to do more than evacuate the blood-clot and ensure adequate drainage, especially where pus is present.

### Gumma of the Cardiac End of Stomach.

The case reported by Cronquist in the *Arch. f. Derm. und syph.* is particularly interesting from the point of view of diagnosis. The patient had been under treatment for a supposed cancer of the cardiac end of the stomach. Œsophageal stenosis had gradually progressed until the patient was finally unable to take anything into the stomach, and only with great difficulty could a sound be passed into the organ. It was only from an accidental conversation with the patient's wife that the doctor in charge of the case came to have a suspicion of its true nature. She told him that at one time her husband had suffered from intense headaches, for which he had been treated by inunction. Suspecting syphilis, the practitioner ascertained that the patient had never had a chancre, but that ten years before he had suffered from intense headaches, with ptosis and paresis of one-half of the face and both lower extremities. All these phenomena persisted for a long time, and only disappeared after inunction for a fortnight with an ointment prescribed by a physician. The patient was therefore treated with mercury and iodides, and in eight days' time was able to absorb liquid food, and six weeks later to swallow solids almost without discomfort. Examination with the sound showed that a certain degree of stenosis still existed, which the author believes to be due to the scars left by the gumma.

### Treatment of Spastic Paralysis.

Foerster, writing in *Mitth. aus. d. Grenzgeb. d. Med. u. Chir.*, calls attention to the treatment of spastic paralysis by section of the posterior nerve roots. Reflex irritability of the cord is lessened by the inhibitory influence exerted by the pyramidal tracts of the spinal cord, a function these tracts possess in addition to that of transmitting motor impulses. If, therefore, these tracts be interrupted, in addition to paralysis and exaggeration of the tendon and skin reflexes, there will be present extensive involuntary movements and spastic contractures, which are reflex and dependent upon sensory irritation in the skin, joints, tendons, muscles, and ligaments. This irritation is conveyed to the cord along the peripheral nerves and posterior nerve roots, and results in the cord responding by contractions of the muscles due to motor irritation. The author, by cutting the posterior nerve roots, proposes to overcome these results. At his suggestion Tietze operated upon two cases of infantile spastic diplegia, one of spastic paraplegia due to Pott's disease, one of hemiplegia, and one of multiple sclerosis. In three cases the results were very encouraging, spasticity being either abolished or much improved, with corresponding improvement in the voluntary movements. In the hemiplegic case the contractures of the arms were lessened, but there was no improvement in the voluntary movements, from which it would seem that the upper extremities are not so amenable to treatment as the lower. The operator advises that the operation should be performed in two stages, at the first of which the dura is exposed, the nerve roots being cut at the second.

